# Keep Calm and Carry on: Parental Opinions on Improving Clinical Dietary Trials for Young Children

**DOI:** 10.3390/nu10091166

**Published:** 2018-08-25

**Authors:** Analise Nicholl, Therese A. O’Sullivan

**Affiliations:** School of Medical and Health Sciences, Edith Cowan University, 270 Joondalup Dr, Joondalup WA 6027, Australia; t.osullivan@ecu.edu.au

**Keywords:** paediatric, dietary trial, intervention development, child-centred research

## Abstract

Recruitment can be an issue for paediatric research. We aimed to investigate parental opinions of paediatric clinical assessments, and to combine findings with recent literature to inform the design of a clinical dietary trial. We used convenience sampling to recruit 17 parents of children aged 2–6 years from two community playgroups in Perth, Western Australia. Three focus groups considered proposed child assessments, study design, and potential study enrolment. Qualitative thematic analysis of focus group transcripts used NVivo 11 (QSR, Melbourne, VIC, Australia). Four main parental concerns emerged, presented here with solutions combining parent responses and relevant literature. (1) Parent and child needle fear: a good experience and a good phlebotomist help keep participants calm, and offering additional analysis (e.g., iron status) makes blood tests more worthwhile. (2) Concerns about children’s age, stage, understanding and ability to cope: create a themed adventure to help explain concepts and make procedures fun. (3) Persistent misunderstandings involving study purpose, design, randomization and equipoise: provide clear information via multiple platforms, and check understanding before enrolment. (4) Parental decisions to enrol children focused on time commitment, respectful treatment of their child, confronting tests and altruism: child-centred methodologies can help address concerns and keep participants engaged throughout procedures. Addressing the concerns identified could improve participation in a range of paediatric health interventions.

## 1. Introduction

It is common for paediatric clinical trials to struggle to recruit enough participants. Ethical and liability concerns, a lower burden of childhood disease, and the fact that children do not react to treatments as consistently as adults all combine to affect both the funding and the numbers available for paediatric trials [1]. As a percentage of total clinical trials, paediatric medical interventions often only reach single digits in neonates and young children, particularly in low-income countries. This has a potentially severe impact on attempts to improve global child health and equity [1]. At the family level, parental reservations around medical type testing of their children are likely to be an important reason. Researchers often develop assessment protocols for paediatric clinical trials with limited community consultation and plans to maintain participant engagement [2]. Good pre-trial qualitative research can explore aspects of a healthcare intervention, generate hypotheses and refine proposed methods [3]. However, there appears to be little reporting on qualitative research to improve the design of clinical dietary trials [3,4]. The failure to publish this kind of research may be due to a lack of resources, limited acceptance by peer-reviewed journals, or poor integration into the peer-reviewed publication of the clinical trial [3,5]. 

Most literature reporting on community consultation for paediatric trials has focused on factors influencing parents’ decisions to enrol their child in a clinical trial [6,7,8,9,10,11,12]. Results from these studies show that parents value practitioners and researchers who consider the needs and preferences of their children [11]. A major barrier to child participation is that researchers and health professionals often seem to marginalize children and their preferences [13]. The benefits of involving a child in the research experience, or in their own care decisions, can include improved service provision; increased child self-esteem, self-expression and empathy; improved sense of belonging; a greater internal locus of control with more responsibility; and increased adherence to regimes, tests and treatments [13,14].

Research directly consulting with parents about proposed methods for paediatric dietary and healthcare interventions is rare. We aimed to use a small community consultation to explore attitudes towards paediatric assessments and procedures, common to both dietary and clinical trials, with due consideration of both the young child participants and their parents as caregivers. Results, in combination with relevant literature, are intended to inform a dietary clinical trial into the effects of dairy fat on child health. Findings may also prove useful to help improve the evidence base for the design of other paediatric health interventions.

## 2. Materials and Methods 

### 2.1. Study Design

Qualitative research is an increasingly popular methodology for community consultation, as it allows for open-ended and flexible ways to explore complex problems [15]. We chose to use focus groups for this study, as they can provide a richness of detail and depth of description not available from quantitative surveys [16]. Individual interviews can elicit detailed information, relatively free of group pressure; however, they are time-consuming and can be restricted by the need for consistency of structure and environment. Focus groups can simultaneously explore multiple viewpoints, group consensus and complex issues arising from discussion; reveal community priorities and language; refine questions and explanations, and provide valuable insights for future participant interactions [17], all in a natural environment [18]. These benefits appeared to outweigh the risks of group pressure in our study design. Involving parents from community playgroups, acquainted with each other and with each other’s children, appeared to provide the best way to gain insightful parental input into study design and assessments that would be respectful of young children. The consultation was performed to inform a proposed double-blinded randomised dietary clinical trial, which aimed to compare the effects of regular versus reduced fat dairy products on cardiovascular and gut health in young children aged between two and six years, using assessments common to both dietary and clinical trials. 

### 2.2. Participants

Parents were recruited via a convenience sample at two community playgroups in Perth, Western Australia. Both these community playgroups are located in an area with the same Socio-Economic Indexes for Areas (SEIFA) rating for socioeconomic status as the area hosting the proposed paediatric clinical trial: both areas score a 9 for relative socio-economic advantage [19]. Inclusion criteria were being the parent of a child currently aged between 2–6 years and able to attend the playgroup venue, or willing to participate in an online focus group. Non-English speaking parents or parents under 18 years were excluded. We decided to use parent focus groups, rather than parents and children, as children in our age group of interest (2–6 years) are not considered to have sufficient language and social skills for focus group participation [18]. The Edith Cowan University (ECU) Human Research Ethics Committee approved the study (Project code: 13931), and all participants provided signed or implicit informed consent. 

### 2.3. Focus Groups

Parents had the option of attending one of two in-person focus groups. Permission was obtained to hold these in or near the playgroup venue, at the same time as the regular group sessions. Parents were welcome to bring their children, and a supervised play area was provided. Parents who could not attend in person were invited to participate in an online focus group, available via a private Facebook page. Online focus groups have been found to be flexible and convenient by parents, and are considered to be a feasible tool for qualitative research in paediatrics [20]. We provided participant information leaflets and an informed consent form to parents who attended in person. Parents in the online focus group had access to an electronic information sheet and implied consent was obtained. A brief sociodemographic survey was also completed. This requested details of participating parents’ age, gender, marital status, education level and employment status; previous family participation in research, and children’s age and gender. It also surveyed their young children’s dairy intake.

In-person focus group sessions lasted around 50 min. These semi-structured sessions were recorded using two Olympus digital voice recorders VN-731PC (Olympus Corporation, Tokyo, Japan). The recruiting researcher (TOS) moderated all groups to provide consistency. This included moderation of the text-based online focus group by posing questions, regularly checking for posts, clarifying answers and encouraging group discussion. TOS queried study misunderstandings when they arose in focus group discussions, and summarised information provided in the parent information leaflets. 

Each focus group considered the proposed child assessments, study design and potential study enrolment via mostly open-ended questions (the topic guide is shown in Box 1). We sought feedback on the following assessments: body composition as measured by the BOD POD air displacement plethysmography system (Cosmed, Rome, Italy), blood pressure, strength testing, dietary assessment, blood tests and faecal samples, We provided pictures of all procedures and equipment to all focus groups. Parents attending the in-person focus groups each received a small gift voucher and morning/afternoon tea as compensation for their travel time. 

### 2.4. Data Analysis 

All focus group recordings were transcribed verbatim (by author AN). Paper-based sociodemographic survey forms were entered into Qualtrics survey software (Provo, UT, USA) by AN; online forms were completed in Qualtrics directly. Playgroup and online focus group data was coded using the qualitative data analysis software package QSR NVivo 11 (Melbourne, Australia), and AN used second order coding to identify parental issues and proposed solutions. De-identified focus group participants were categorised by gender and first participation in the relevant focus group, e.g., female parent 2 (FP2), focus group 1 (FG1). Themes and sub-themes were identified by larger numbers of coded statements cumulatively associated with each concept, and then refined via second-order coding. TOS conducted a smaller thematic analysis, using paper-based methods to identify themes from the focus group transcripts. This provided an independent investigator and method analysis triangulation of the data. AN and TOS subsequently achieved consensus on the inclusion or exclusion of themes and subthemes via researcher discussion.

Box 1 **Topic guide for parental focus groups.**

**Figure 1:**
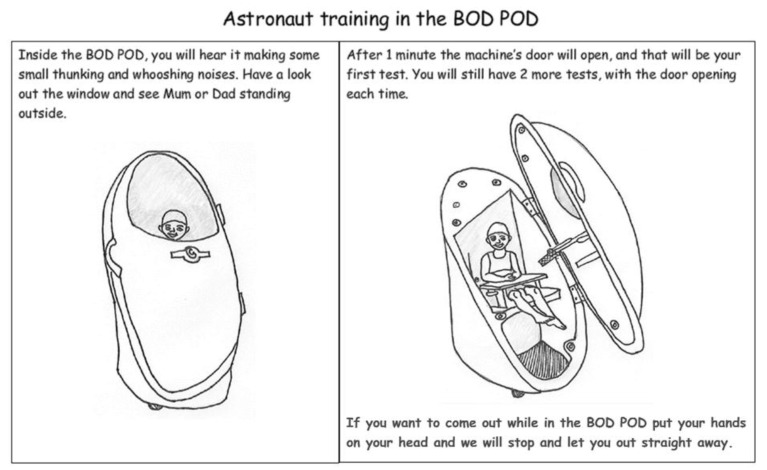
Excerpt from our child-friendly information leaflet: Astronaut training in the BOD POD. The BOD POD uses non-invasive air-displacement to measure body composition (lean mass and fat mass). Participants need to sit inside during testing, and there is a paediatric seat for children under six years of age (Page reproduced from the Milky Way Study Child Information Leaflet. Pictures by Julie Hill, Student, Masters of Nutrition and Dietetics, Edith Cowan University, Western Australia).

- Introduction to the proposed dairy study
  ○ Why it is important
  ○ Overview of what would be involved
- Brief explanation of assessments planned and what information they provide
- Specifically seeking parental opinions on:
  ○ Blood measures—fasting
    ■ Preferred location for sampling—home or hospital/clinic?
    ■ Analysis for cholesterol and triglycerides planned—would parents be interested in having additional tests done for their own benefit?
    ■ Concerns around safety or child anxiety, ideas to minimise
  ○ Body composition
    ■ Any preference for BOD POD *, DEXA ** or BIA ***
    ■ Concerns around safety or child anxiety, ideas to minimise
  ○ Stool samples
    ■ Thoughts on feasibility of collecting samples
    ■ Storage preferences—at home, collected by research assistant or stored in mini-freezer provided by study at home
  ○ Body strength
    ■ Would a child be capable and willing to do the test procedure (isometric thigh pull)
  ○ Dietary assessment
    ■ Thoughts on feasibility of keeping 3-day food records for child
  ○ Dairy products
    ■ Delivery methods
    ■ Information required on labels
- Any further thoughts to improve the study experience for parent or child?
- Would you enrol your child—why or why not?

* The BOD POD uses non-invasive air-displacement to measure body composition (lean mass and fat mass). Participants need to sit inside during testing, and there is a paediatric seat for children under six years old. A diagram is shown in Figure 1. ** DXA: dual-emission X-ray analysis. Comparison of differences when X-ray beams pass through bone, lean tissue and body fat is used to estimate the relative mass of each. DXA exposes patients to radiation. *** BIA: bio-impedance analysis uses low-level electrical currents and leads to measure the electrical impedance of body tissues, allowing estimation of lean mass and fat mass.

## 3. Results

### 3.1. Participants 

From the 23 parents invited, 17 parents participated in one of the three focus groups. Three parents attended the first focus group (1 male), five participated in the second (0 male), and eight contributed to the online focus group (2 males). The majority of parents were mothers (*n* = 14; 82.4%). Of the 15 parents who completed the sociodemographic questionnaire, the majority were married (86.7%), university educated (86.7%), working part time (53.3%) and had an average age of 36 years (range 33–41 years). All parents had at least one child in our age range of interest (2–6 years); all these children drank regular-fat milk and regularly ate cheese, yoghurt and butter. Four parents (26.7%) reported that they or their 2- to 6-year-old child(ren) had previously participated in a clinical trial.

### 3.2. Thematic Analysis of Focus Group Data: Major Parental Concerns 

Parents were invited to comment on each of the proposed trial assessments, improving the dietary trial as a whole, and then as to whether or not they would enrol their child in the trial if they had the opportunity. Convenience sampling and parenting constraints limited our ability to achieve data saturation, particularly around exploration of minor concerns. Four major themes emerged. These are shown with representative parent quotes in Table 1. 

#### 3.2.1. Theme 1: Parent and Child Needle Fear

This was the most dominant issue in all three focus groups. Some parents found that their own childhood anxieties and bad experiences with blood tests (or immunization ‘needles’) inclined them against blood tests for research purposes. A previously unconsidered issue emerged: that parent fear might also need to be managed.

#### 3.2.2. Theme 2: Child’s Age, Stage, Understanding and Ability to Cope

Arising during discussions of the BOD POD body composition analysis (a brief description is given in the footnotes for Box 1, and a representation in Figure 1), body strength testing and the issue of blood samples, this theme related mostly to children’s anxiety and their ability to keep still and/or to follow instructions. The additional issue of child age of informed assent was also mentioned. 

#### 3.2.3. Theme 3: Study Misunderstandings

We encountered persistent misunderstandings involving the purpose, design, randomization and equipoise of our planned dietary clinical trial in all three focus groups. Prior beliefs, the views of other parents and misinterpretation of public health guidelines all appeared to contribute to the following misunderstandings: 

(i) Some parents were not aware that the dairy guidelines call for reduced fat dairy for children over the age of 2 years. This proved constant across all three focus groups, in spite of written and introductory information that one of the purposes of the proposed dietary trial is to better inform the Australian Dietary Guidelines’ recommendation. This then paved the way for misunderstandings ii and iii.

(ii) Regular fat dairy is healthier, as reduced fat forms are full of added sugars: a belief that reduced fat dairy has potentially harmful additives, such as sugar, to replace the fat interfered with attempts to explain randomisation and equipoise.

(iii) Equipoise randomisation to the reduced fat dairy group will disadvantage the child: parents needed repeated reassurance that we would not knowingly disadvantage their child by placing them in this arm of the study. This persisted in spite of explanations that ethics forbids known disadvantage to any group of trial participants (equipoise).

#### 3.2.4. Theme 4: Factors Affecting Parental Decisions to Enrol Their Children in the Study

Negative influences included trial time commitments; inconsiderate or harmful treatment of their child (the term ‘guinea pig’ was used); and confronting tests. Altruism was the major positive influence. Some parents flagged that they did not want any dairy packages opened during blinding of dairy products, for food safety reasons. This led to re-evaluation of proposed blinding procedures. 

### 3.3. Proposed Solutions 

Parents proposed potential solutions to some issues raised during the focus groups, such as improving childhood anxiety. Misunderstandings of study purpose and design proved difficult to correct at the time, and proposed solutions came later from researcher discussions and literature review. This combination of parent-, literature- and researcher-derived solutions is presented in Table 2. 

#### 3.3.1. Theme 1: Parent and Child Needle Fear: 

• **A Good Experience and a Good Phlebotomist Trump Convenience or Location**

Location preferences varied between having tests at home, at the city’s specialized children’s hospital or at a local pathology centre branch. However, the manner and competence of the phlebotomist soon emerged as the most important factor. Parents all agreed that they wanted their child to have a good experience and not to be scared of future testing. Suggestions to improve the experience included the use of numbing gel and a teddy bear or toy demonstration, to assist child understanding. 

• **Additional Analysis of Samples Acts as an Incentive**

Parents considered additional testing of the blood samples to be a good incentive to have the sample taken. Opinions favoured iron studies and vitamin D testing, as most felt they would not otherwise subject their children to these without a doctor’s request. Several parents, particularly mothers with personal experience of iron-deficiency, were positively swayed towards the blood tests by this incentive. 

#### 3.3.2. Theme 2: Child’s Age, Stage, Understanding and Ability to Cope: 

• **Create a Themed Adventure to Help Explain Concepts and Make it Fun**

Ideas for themed adventures centred on the BOD POD body composition capsule (represented in Figure 1) looking like a rocket ship (space theme) or a large egg (dinosaur theme). Ideas included dress-ups; rewards; softening stark clinic rooms with decorations; animated research assistants; and adding background music. A video or games on the iPad (or digital device) was suggested to help keep children still during BOD POD analysis. 

#### 3.3.3. Theme 3: Study Misunderstandings: 

• **Correct Parent Misunderstandings before They Enrol Their Child**

This conclusion was reached after all three focus groups exposed the same study misunderstandings. Parents need simple explanations in clear language, but it is not enough just to provide written information on enrolment. Making information available via a variety of platforms, including online, and providing ample opportunity to gauge parent understanding verbally can help alleviate their concerns. Providing relevant product nutrition when responding to early expressions of interest might help prevent misunderstandings specific to a dietary trial.

#### 3.3.4. Theme 4: Factors Affecting Parental Decisions to Enrol Their Children in the Study: 

• **Use Child-Centred Research in Study Design to Help Address Concerns and Keep Participants Engaged Throughout**

Parents expressed the importance of consideration of their children in research. They believed that the majority of issues raised could be approached using basic principles of child-centred practice, including building rapport and trust, appropriate assessment tools and making the process enjoyable. 

## 4. Discussion

We aimed to investigate parental opinions on a planned paediatric clinical dietary trial, and to combine a small community consultation with relevant literature to optimize the experience for participants and their parents. Four major themes emerged from the thematic analysis of the focus group data. 

### 4.1. Parent and Child Anxiety 

The first two themes, parent and child needle fear and parental concern about their child’s age, stage, understanding and ability to cope, took up the major part of focus group discussions. Blood tests are invasive, and can be both painful and frightening. Needle fear is most commonly caused by vaccinations; it generally appears at around 5–10 years of age, by which time children may have had more than a dozen vaccines [23]. If left untreated, it can not only affect adult treatments but can also be modelled to the next generation. Effective anaesthetic creams and numbing gels are now routinely available [22], and consistent and appropriate pain management should be used during all childhood needle procedures [23]. The use of numbing gel was endorsed by a number of our focus group parents. However most research into this area is quantitative, and without qualitative input from participants themselves solutions may be inadequate, or inadequately presented [29]. We plan to use summative evaluation by clinical trial participants and their parents to refine our findings. 

During blood tests the performance and behaviour of health professionals have been found to correlate more strongly with the child coping well, while parents’ behaviour correlated more strongly with child distress [21]. In a study that video-recorded the responses of 50 children and their carers to venepuncture, parental reassurance emerged as the strongest factor promoting child distress, above criticism [21]. While this seems counter-intuitive, well-meaning reassurance affected by parent childhood experiences could alert the child that there is something to fear. It appears that the healthcare practitioner may have the strongest ability to help manage child distress, in part by promoting coping behaviour in parents as well [21].

Non-medically prescribed child blood tests can be regarded by parents as an inconvenience, and add to the study burden of participation [12]. However, additional analyses (e.g., child iron status) proved an acceptable incentive to many parents to have blood samples taken. The use of incentives in paediatric research trials is controversial, as they may distort decision-making and create selection bias; however, guidelines outlining acceptable incentives are often inconsistent [22,30]. Blood volume needs to meet strict guidelines for the quantity of blood ethically able to be drawn from a young child, based on their weight and on demonstrated research needs [31]. If used, incentives should be appropriate for the age of the child concerned; be acceptable to the relevant research ethics committee; and information and choices should be clear to parents [22] through parent information leaflets and informed consent forms.

Concern around children’s age, stage, understanding and ability to cope related mostly to anxiety and their ability to keep still and/or to follow instructions. Collaborative focus group discussions identified a practical set of solutions: create a themed adventure, with toys, dress-ups, background music, rewards, and use of an iPad to reduce child anxiety. French research on 115 children aged 4–10 years has shown that the iPad (*n* = 60) was at least as effective as a sedative (midazolam, *n* = 55) at keeping both children and their parents calm while waiting for paediatric surgery [24]. However, its use during a procedure must be weighed up against the potential to distract children from listening to instructions and understanding what is happening to them during the procedure. 

The issue of the age of the child also affects when informed assent can be considered appropriate. As highlighted in Table 1, one of our focus group parents would not consider enrolling a child under 5 years old, as he felt the child would need to understand what is going on in order to give verbal informed assent. In children, true ‘informed assent’ takes into account the child’s age, ability to process information relating to their health condition and emotional readiness for this process [32]. The general opinion appears to be that seven years old is a suitable minimum age of informed assent [8,13,22,33], with progressively more competent decisional reasoning expected during adolescence [33]. 

For ethical reasons, paediatric qualitative research should consider the voices of the children themselves. However, young children are too often considered to be developmentally immature; hence researchers often seek proxy information about them, giving rise to the so-called ‘missing child’ paradox [34]. We found limited research consulting children directly [9,13], and none that consulted children below seven years. Our own playgroup research did not directly involve the children present, because children under six years are considered too young to have sufficient language and social skills for participation in focus groups [18]. Evaluation forms for the children to rate their experiences during piloting of methods may help to overcome this limitation, and is discussed further below.

### 4.2. Study Misunderstandings and Other Factors Affecting Parental Enrolment

Parents misunderstood some aspects of our proposed clinical trial design, purpose, randomization and equipoise. There is little consensus on best practice for recruitment of children into clinical trials and obtaining informed parental consent [10]. Among study misunderstandings identified in previous research, randomization and participation practicalities were factors found to affect parental decision-making [10], with some similarity to our findings. The specific belief that dairy guidelines call for regular fat dairy for all children persisted in all three parent focus groups, in spite of written and verbal information that one of the study purposes is to better inform the national guidelines. The Australian Dietary Guidelines for Children recommend consuming mostly low fat dairy after two years of age [25], and the proposed dietary clinical trial aims to test this assumption on the basis of recent evidence. 

Most focus group parents believed that regular fat dairy is healthier, because reduced fat forms were thought to be ‘full of harmful additives, in particular added sugars’. As a result, we decided to provide advance nutrition information (other than fat and energy content) on all dairy products to be used in the trial, to show that our selected reduced-fat products have similar sugar content to the regular-fat versions. Focus group parents persisted in the negative belief that randomisation to the reduced fat dairy group could harm their child’s health. This was in spite of noting that we could not ethically disadvantage any child by placing them in either arm of the study (equipoise).

Additional negative influences affecting parental enrolment into our planned dietary trial included study time commitments (burden of participation) [10]; a fear of careless or harmful treatment of their child [12]; and confronting tests. On the other hand, altruism was a major positive factor [6,7,9,10,26] (Table 1). The concern that children are treated like ‘guinea pigs’ in clinical trials is longstanding [1,12]. As we found, parent decision-making to enrol their child in a clinical trial can be a complex process, involving constant risk–benefit analyses around parent, child and study characteristics [6,7,8,12]. Factors affecting enrolment decisions need careful consideration, in order to improve historically low rates of participation in paediatric clinical research [1]. Interest in the delivery, blinding and personal labelling of our proposed study dairy products during the three-month intervention appeared to support advertising free dairy as a positive incentive for enrolment. 

To address above parental concerns, information on study purpose, randomization and equipoise should be clearly communicated in interviews, parent information leaflets and informed consent forms, with time allowed for questions before parents sign consent [10,11]. Interactions at recruitment offer opportunities to tailor discussion to the needs and situations of individual parents [26]. Practical solutions to improve recruitment and within-trial engagement include: clear written information; improving parent discussion with open-ended questions scaffolded around particular trial issues, and answering within-trial questions, e.g., with online forums and social networking [10]. Within-trial engagement could also include appropriately-timed reminders of the purpose of the trial to encourage data collection; showing appreciation for all contributions, and listening to parent and child experiences in order to inform later trials [2]. A study Facebook page, or similar networking website, which can offer information, progress details and opportunity for comment, would suit this purpose. 

Our belief that young children might need additional considerations, planning and resources within a healthcare intervention appears to be relatively novel in the paediatric dietary and clinical trial literature. Our study conclusions promote a whole-of-intervention approach incorporating child-centred research, as outlined in the following section. Accordingly, in our study clinics, all procedures and child assessments have been planned to include evaluation by both parents and children, with the aim of informing a larger dietary trial. 

### 4.3. Child-Centred Research 

Parents value consideration of the needs and preferences of their children [11]. Child-centred approaches are increasingly valued in both care and research. Although parents should always be considered an authority on their own children [29], there is a need for behavioural models that show respect for children and allow some participation in decisions that affect them. 

One such model is Shier’s Pathways to Participation, which recommends introducing five increasing levels of participation: (1) that children are listened to; (2) supported when expressing their views and opinions; (3) have their views actively considered; (4) are involved in the process of making decisions; and (5) share the responsibility of making decisions that affect them [14]. However, at 2–6 years the children in our planned research are younger than the seven years considered the minimum for true informed assent, as discussed above. We investigated a model developed by childcare specialist Magda Gerber, Resources for Infant Educarers (RIE) [27], which includes three particularly relevant principles:

1) Use authentic communication (honest explanations about why a test is needed, and what to expect, can empower the child).
2) Acknowledge emotions (acknowledge the child’s feelings are valid, including fear).
3) Invite participation (children can practice on a teddy bear or toy before the assessment, or help set up machines or equipment where suitable) [28].

A research assistant dedicated specifically to observing, building a relationship and working with the children at each research clinic visit could ensure practical application of these principles during paediatric clinical and dietary trials, and this has been a major focus of our child-friendly planning.

Evaluation and improvement of procedures is important, particularly at the pilot stages of a trial. Asking young children to describe symptoms or emotions may be beyond their capacity, and the use of careful observation and tools such as emoticon Likert scales and face pain scales may be more appropriate [1,23]. Young children do remember previous painful procedures, and they are capable of assessing, evaluating their reactions, and suggesting improvements [29]. This may then help create a more positive experience, with an improved likelihood that they will understand and participate in subsequent procedures [29]. We aim to trial child-centred, participatory evaluation tools for young children in our clinical trial, including photographs and emoticon-face Likert scales.

**Figure 2:**
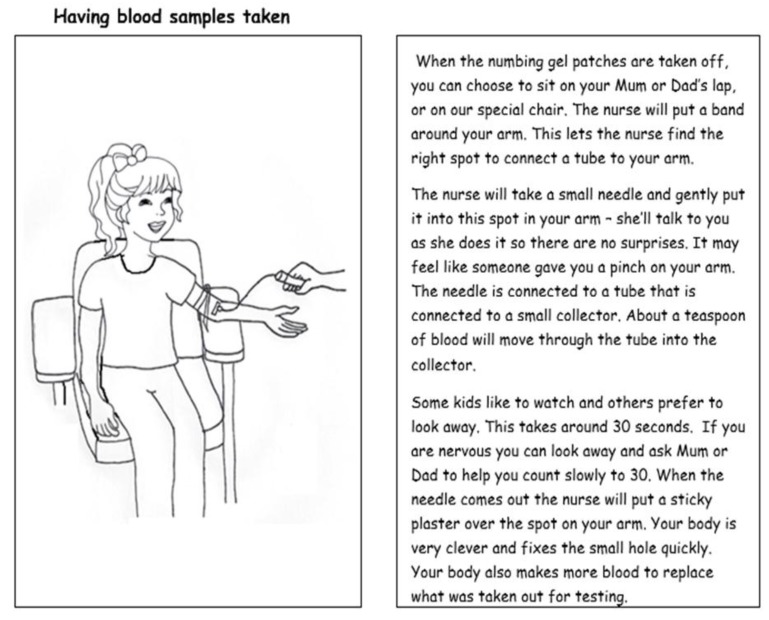
Excerpt from our child-friendly information leaflet: Having blood samples taken (Page reproduced from the Milky Way Study Child Information Leaflet. Picture by Julie Hill, Student, Masters of Nutrition and Dietetics, Edith Cowan University, Western Australia).

**Figure 3:**
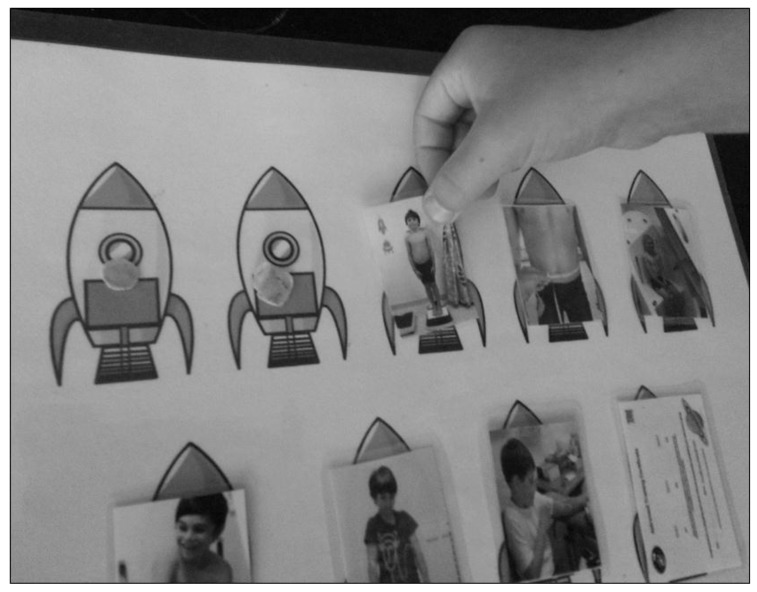
Child-friendly resource showing the sequence of tests at a trial clinic visit, with detachable photographs able to serve as a visual guide both to test procedures and to track progress over the visit: from left to right photographs present represent assessment of weight, waist measurement and BOD POD body composition analysis (top row); blood pressure, strength, blood test and ‘graduation’ certificate awarded after all clinics completed (lower row, cut off), which are mostly common tests in clinical trials. (Poster and photographs designed for the Milky Way Study by Kate Evelegh, Student, Masters of Nutrition and Dietetics, Edith Cowan University, Western Australia. Copyright and permissions: K. Evelegh and the Milky Way Study).

Providing a pictorial child information leaflet can help explain all research procedures to the child in advance, and it can be tailored towards educating the whole family [12]. Examples from the Child Information Leaflet for our clinical trial show our suggested, space-themed approach to ‘astronaut training’ in the BOD POD and blood tests in Figure 1 and Figure 2. Another child-centred research tool could be a poster with a detachable sequence of photographs for all clinical assessments. This acts to provide the child with an idea of the schedule for the session, and removal of photographs after each procedure would then serve as a visual guide to progress over the session. An example is shown in Figure 3.

### 4.4. Strengths and Limitations

Limited sample size is often a concern in qualitative research [15], and our limited number of participants and recruitment settings risked selection bias [20] and failure to achieve data saturation [35] with some of the minor themes. These included a few of the lesser assessments, dairy deliveries and food/sample storage, as proposed in the focus group topic guide (Box 1). Our participants all displayed similarities in age, stage of life, education and socioeconomic status. The location of our in-person focus groups and the location of our study were very similar, both scoring a SEIFA decile of 9 for relative socio-economic advantage and disadvantage. This reflects a high level of advantage in both areas. We consider the location of the focus group to be similar to the study location. However, for dietary trial recruitment we decided to focus on a broader range of suburbs, within 30 km of our study’s university location. Our use of playgroups to recruit a convenience sample of parents may have attracted parents more inclined towards clinical research [15], as one in four of these focus group families had previously participated in a clinical trial. However, one of the target groups for the paediatric trial is the university networks, which include parents potentially well-disposed towards research These are all factors that need to be considered in the transferability of our results to the broader community. 

It emerged that group interaction between parents who meet regularly at a playgroup for their children created an environment where parents felt comfortable to share new and unanticipated ideas, priorities and practical suggestions [17], providing a richness of detail and depth of description not available from quantitative surveys [16]. Independent data triangulation of the focus group transcriptions helped assure the quality of the thematic analysis by preventing coding bias. Combining a local community consultation with appropriate solutions from the literature gave us essential insight into the design of a practical and respectful paediatric dietary intervention. 

## 5. Conclusions

Results of this research may assist in planning a range of child dietary and health interventions. Qualitative research that considers the personal experiences of participants could improve clinical trials by reducing the burden of participation and achieving better compliance. It may also help to recruit and retain larger numbers of participants in paediatric clinical trials. Parents appear to value child-centred research. Providing a respectful and fun experience for children during clinical assessments can help keep both children and parents calm and enable the study to carry on.

## Figures and Tables

**Table 1:** Major parental issues identified in two in-person focus groups (FG) and one online focus group (OFG) (*n* = 17). De-identified focus group participants were categorised by gender and first participation in the relevant focus group, e.g., female parent 2 (FP2), focus group 1 (FG1).

Issue	Example Parent Quotations (Parent Code (M/F Parent) and Focus Group Reference)
Theme 1: Parent and child needle fear	*I don’t know. I just don’t, um, see—except if it’s for a medical reason, I suppose ... I hate blood tests at the best of times, so to subject my 3-year-old to it, as well? It’s going to become like World War II ...* (FP3, FG2)
	(Child’s name) *and I were part of an egg allergy study last year and he had to have blood taken three times. It was a very challenging experience for me.* (Child’s name) *cried and cried through the blood taking* (FP3, OFG)
	*Keeping them* (the children) *calm is the main problem* (FP5, FG2)
Theme 2: Child’s age, stage, understanding and ability to cope	*Yeah, probably—probably for a 5- or a 6-year-old I might, but I don’t think I’d try and do it for a 2- or a 3- or a 4-year-old ... Just the trauma of it, and they don’t—not sure that they’d quite understand ... Yeah, when you can actually ask them whether they’re willing to participate in it—until then ...* (MP1, FG1)
Theme 3: Study misunderstandings (i) Lack of awareness of current dietary guidelines promoting low-fat dairy consumption for children over 2 years	*I’m like, I just thought it was, like, you always feed your kids fat—it’s not until you are an adult that you ... I didn’t realize that was the official recommendation?* (FP2, FG2)
(ii) Regular fat dairy is healthier, as reduced fat forms are full of added sugars:	*I thought full fat dairy was recommended too. I thought low fat in dairy tends to have high sugar, and sugar is far worse than fat.* (FP5, OFG)
(ii) Equipoise: randomization to the reduced-fat dairy group will disadvantage the child	*See, I don’t, I’m not really that keen on giving them low-fat dairy for 3 months, really, particularly because my girls are, um, already at the bottom of the charts as far as, um, percentiles—particularly* (child’s name)*. She’s, like, already quite a skinny baby?* (FP2, FG2)
Theme 4: Factors affecting parental decisions to enrol their children in the study Negative: time commitment; treatment of their child; confronting tests Positive: altruism	*Um, to be honest, probably not ... Just because of the commitment that’s required? Um, I’m for it, I’m all for getting it—get the study done, but in terms of—because I’ve got a little one and a whole lot of other commitments, and to be able to manage my time and ... I guess, you know, getting a blood test—I mean, if it’s ... for the better good, I would do it, but I’m - inclined towards not going there - mainly for commitment reasons, then, partly for scare - you know, scaring off my little kids ... Mostly the blood test, yeah, that would be the ... I guess it’s, you know, trying to keep on top of it with all the, um, the food that’s coming in, and tracking it, and, um, the stool, yeah. So that’s quite a lot of things to do ...* (FP1, FG1)
	*It’s difficult to know whether to participate. For example, the egg allergy survey: we did all the tests but not the dietary changes because we wanted to introduce eggs earlier (than the study prescribed). (*Conflicted*) They need people, but the conditions? They treat (children) like guinea pigs.* (FP1, FG2)
	*I don’t mind the dairy (part of the trial). (I’m not so sure about the) poo thing ...? (With the) blood tests, I would want my child to be older–two-and-a-half (years) plus.* (FP5, FG2)

**Table 2:** Parent, literature and study solutions to issues identified in focus groups. De-identified focus group participants were categorised by gender and first participation in the relevant focus group, e.g., female parent 2 (FP2), focus group 1 (FG1).

Issues Identified	Proposed Solution(s)	Example Parent Quotations Quotation, Parent Code (M/F Parent) and Focus Group (FG; OFG = Online Focus Group)	Support from the Literature
Theme 1: Parent and child needle fear	A good experience and a good phlebotomist trump convenience or location	*As long as it’s somebody that’s highly experienced with children, because taking (blood) from your child is a completely different thing—they really don’t have any control. It’s that fear, it’s that result*. (FP2, FG2)	The healthcare professional may be the best person to manage a child’s distress, in part by helping parents cope [21]
		*My daughter had quite a few blood tests when she was 4, the numbing gel worked well for her. The blood tests she had were taken at* (the major children’s hospital in Perth). *The staff were fantastic and very good at distraction, using things like bubbles, and showing her colorful pictures* etc. *Our job was to try to keep her still and as calm as possible, They also had everything in place and ready before taking the blood. She still got upset, but the gel made it less painful and distraction made it a bit easier.* (FP3, OFG)	Effective anaesthetic creams and numbing gels are now routinely available [22], and consistent and appropriate pain management should be used during all childhood needle procedures [23]
		*I think the numbing gel definitely. I like the idea of the children practicing on the teddy or doll first only cause our kids are a just a little bit older. Not sure how* (child’s name) *would go cause she’s younger. I guess it would be a case of applying the gel distracting her until the gel kicks in and giving lots of cuddles after* (MP1, OFG)	
	Additional testing of samples acts as an incentive	*So, with the blood, I have a, like, genetic thing. Being Mexican it’s quite common for a large percentage of ... women to be anemic. That’s something that’s worth looking for. So, I know I need an injection of iron or an IV infusion every 6 months or so. I do often wonder about my girls—something about what they need ... (If) they don’t, I will try to give it to them—like meat, specially, and stuff like that ... I find it hard to get kind of enough iron into them—most of the time, so I do wonder—do they always get enough iron in their diet?* (FP2, FG2)	The use of incentives in paediatric research trials is controversial, but they should always be appropriate for the age of the child concerned; be acceptable to the relevant research ethics committee; and information and choices should be clear to parents [22]
Theme 2: Child’s age, stage, understanding and ability to cope	Create a themed adventure to reduce child anxiety and make it fun	*Yep I think the adult going first (where possible) is a good idea to show the children that it’s not scary* etc. *The iPad or some sort of game is a great idea. My kids have a Nintendo DS. Love the rocket ship idea. Tell them that they’re going on a trip or something.* (MP1, OFG)	iPad games have been shown to be at least as effective as sedatives in reducing anxiety in children and their parents before surgery [24]
		*I think this would be a challenge for my 2 (and-a-) half-yr-old boy, not so much sitting in it, but the fact of keeping still for 2–3 min. Both my 2- and 8-year-old love Star Wars, so probably making it into a game, or definitely dressing it up would help … Yes I think an iPad video would help with them being able to sit still.* (FP4, OFG)	
		*Or, on the day it could be different for (*child’s name*), like he could just, you know, be inconsolable, or he could think it’s an adventure. But if maybe he had peers around at the same time, that—do you know what I mean?—line up, then, perhaps—yeah, like the adventure-seeker to go first? So you are not going to have a problem, then, once there has been a beautiful start of it.* (FP3, FG2)	
Theme 3: Study misunderstandings	Correct parent misunderstandings before they enrol their child		
(i) Lack of awareness of current dietary guidelines promoting low-fat dairy			Australian dietary guidelines for children recommend mostly low fat dairy after two years of age [25]
(ii) Regular fat dairy is healthier as reduced fat forms are full of added sugars	Provide nutrition information on all dairy products to parents in advance, to show that reduced fat products have similar sugar content to the regular fat versions		
(iii) Equipoise: randomization to the reduced fat dairy group will disadvantage the child			Provide clear information on randomization, equipoise and that no child can knowingly be disadvantaged in interviews, parent information leaflets and informed consent forms; allow time for questions before parents sign consent [10,11,26]
Theme 4: Factors affecting parental decisions to enrol their children in the study time commitment; treatment of their child; confronting tests; altruism	Use child-centred research in study design to help address concerns and keep participants engaged throughout		Parents have reported valuing practitioners and researchers who consider the needs and preferences of their children [11] Shier’s Pathways to Participation is a child-centred model that recommends introducing five increasing levels of participation: children are listened to; supported when expressing their views and opinions; have their views actively considered; are involved in the process of making decisions; and share the responsibility of making decisions that affect them [14]
			Magda Gerber’s Resources for Infant Educarers (RIE) model promotes respectful treatment of very young children. Three RIE principles, (1) use of authentic communication, (2) acknowledging emotions and (3) inviting participation [27,28] appear particularly applicable to paediatric clinical trials involving young children.
			Research involving children needs appropriate and ethical information provided for recruitment and consent, and should be tailored towards educating the child as well as the family [12]. An example could be to provide pictorial child information leaflets, along with parent information leaflets.
